# Overexpression of BNIP3 in renal carcinoma cells can promote apoptosis of renal carcinoma cells through HIF-1α-BNIP3-mediated autophagy

**DOI:** 10.3389/fonc.2025.1614378

**Published:** 2025-08-05

**Authors:** Long Huang, Lin Wang, Dan Yuan, Yan Xu, Yu Wang, Kai Yao, Xiao Zhong, Quanda Liu, Kang Jia, Lei Lei, Haiyan Wang, Dongliang Liu

**Affiliations:** Department of Urology, 363 Hospital, Chengdu, Sichuan, China

**Keywords:** renal cell carcinoma, BNIP3, B-cell lymphoma-2, HIF-1α, autophagy, apoptosis

## Abstract

**Background:**

Renal cell carcinoma (RCC) is a prevalent malignancy with limited effective therapies, necessitating novel molecular targets. BNIP3, a pro-apoptotic protein regulated by hypoxia-inducible factor 1 (HIF-1), is implicated in autophagy and apoptosis, but its role in RCC under hypoxic conditions remains underexplored. This study investigates the effects of BNIP3 overexpression on RCC cell behavior and its molecular mechanisms.

**Methods:**

Human RCC cell lines A498 and 786-O were transfected with pcDNA3.1-BNIP3 to overexpress BNIP3 and cultured under normoxic (21% O_2_) or hypoxic (1% O_2_) conditions. Proliferation, invasion, and apoptosis were assessed using CCK-8, cell cloning, Transwell, and flow cytometry assays. Autophagy was evaluated via immunofluorescence, transmission electron microscopy, and Western blot analysis of LC3B and p62. Co-immunoprecipitation examined Bcl-2/Beclin1 interactions. In vivo tumor growth was studied using BALB/c nude mice with 786-O xenografts.

**Results:**

BNIP3 overexpression significantly reduced proliferation and invasion while increasing apoptosis in A498 and 786-O cells (P<0.01). Under hypoxia, BNIP3 disrupted the Bcl-2/Beclin1 complex, enhancing autophagy by increasing LC3B and autophagosome formation and decreasing p62 (P<0.01). Autophagy inhibitor 3-MA suppressed BNIP3-induced apoptosis, indicating autophagy-dependent apoptosis. *In vivo*, BNIP3 overexpression decreased tumor volume, Ki67 expression, and increased apoptosis and autophagy markers (P<0.01).

**Conclusion:**

BNIP3 overexpression inhibits RCC progression by promoting HIF-1α-mediated autophagy and subsequent apoptosis under hypoxic conditions, primarily through disrupting the Bcl-2/Beclin1 complex. These findings establish BNIP3 as a potential therapeutic target for RCC, warranting further investigation into autophagy-based interventions.

## 1 Introduction

Kidney cancer is a prevalent form of malignant neoplasm, accounting for approximately 3% to 5% of all adult malignant tumors. Its incidence rate ranks third among male urological malignancies, following only prostate and bladder cancers, with a notably higher prevalence in males compared to females (1, 2). Kidney cancer, which has imposed a significant burden on the health and well-being of individuals globally, currently has an undefined etiology, with smoking, obesity, and hypertension acknowledged as established risk factors (3, 4). Histologically, renal cell carcinoma (RCC) accounts for the vast majority (90%) of kidney cancer cases, mainly including clear cell renal carcinoma (70%), papillary renal cell carcinoma (10%~15%), and chromophobe renal cell carcinoma (5%) (5). Nephrectomy alone is an ineffective treatment, so systemic therapy is imperative for these advanced and metastatic kidney cancers (6). Therefore, there is a need for a deeper understanding of the molecular biological basis of renal cell carcinoma providing new goals for the purpose of diagnosis and treatment of clear cell renal carcinoma. Hence, it is imperative to investigate the mechanisms underlying the progression of renal cell carcinoma and discover novel targets for therapeutic intervention.

BNIP3, a protein involved in promoting cell death, is subject to regulation by hypoxia-inducible factor 1 (HIF-1) (7). Under hypoxic circumstances, BNIP3 could potentially be activated by HIF and subsequently play a role in hypoxia-induced cell death via mechanisms like apoptosis, necrosis, and autophagy (8). It has been pointed out that upregulation of BNIP3 can trigger autophagy (9). BNIP3, via its BH3 domain, exhibits competitive interaction with binding sites on BCL-2 proteins, thereby facilitating the liberation of Beclin-1 molecules (10). Under hypoxia conditions, this process initiates the autophagy mechanism, which in turn affects the survival state of cells.

Autophagy is a highly conserved process that maintains cell homeostasis during evolution, which has a two-sided effect on tumorigenesis and development: on the one hand, autophagy can improve the tolerance of tumor cells to external stress; conversely, triggering autophagy may lead to the promotion of tumor cell death, thereby inhibiting the growth of tumor cells (11–13). By affecting the expression of autophagy signaling pathway-related proteins, the programmed cell death of cancerous cells can be regulated, and the proliferation of tumor cells can be inhibited (14). In the current study of RCC, the improvement in autophagy has an inhibitory effect on the malignant biological behavior of tumors (15).

Based on the previous findings of this study, the expression level of BNIP3 is low in kidney cancer cell lines (8), and based on this, this project intends to construct BNIP3-overexpressing kidney cancer cells to explore whether HIF can interact with BNIP3 to mediate the occurrence of autophagy and affect the growth and programmed cell death of malignant cells.

## 2 Materials and methods

### 2.1 Cell culture and transfection

Human RCC cell lines A498, 786-O, CAKI-1, ACHN, and GRC were purchased from KeyGEN Company (Nanjing, China). These cell lines are well-established models in RCC research and have been widely used in previous studies, enabling better comparison and validation of our results with the existing literature; they represent different subtypes of RCC, which helps us comprehensively understand the role of BNIP3 in various RCC phenotypes, and they also have different levels of endogenous BNIP3 expression. Cells were cultured in Roswell Park Memorial Institute (RPMI) medium using 1640 complete medium (Gibco; Thermo Fisher Scientific, Inc., Waltham, MA, USA). Cells were cultured under hypoxic conditions. Specifically, they were placed in a 3131 Thermo Forma Water-Jacketed CO_2_ incubator and exposed to a gas mixture composed of 1% oxygen, 5% CO_2_, and 94% N_2_ for 48 hours.

PCR was employed to enhance the entire coding segment of BNIP3. The DNA segment was incorporated into the pcDNA3.1 vector (Invitrogen, USA) to produce pcDNA3.1-BNIP3 (pc-BNIP3). An unoccupied pcDNA3.1 vector was employed as a reference (pc-NC) for transfection objectives. Cell transfection was carried out using Lipofectamine^®^ 2000 transfection reagent (Invitrogen, USA). A498 and 786-O cells were seeded into 6 well plates and transfected when they reached a confluence of 70%-80%. To overexpress genes, pc-BNIP3 (2 μg/well) or pc-NC (2 μg/well) along with the transfection reagent (12 μL/well) were mixed separately in serum-free Opti-MEM for 5 minutes. The diluted plasmid and transfection reagent were then combined and incubated at 37°C for half an hour. The resulting mixture was added to the cell culture medium, followed by replacement with fresh RPMI 1640 after six hours of transfection. Culture continued for another two days thereafter.

A498 and 786-O cells were cultured under two different oxygen conditions: cell hypoxia conditions and normoxia conditions. In the cell hypoxia conditions, the gas composition in the incubator was set to 1% O_2_, 5% CO_2_, and 94% N_2_. In the normoxia conditions, the gas composition was 21% O_2_, 5% CO_2_, and 74% N_2_. A498 and 786-O cells were divided into 6 groups: OE-NC (pc-NC); OE-BNIP3 (pc-BNIP3); OE-NC Normoxia; OE-NC Hypoxia; OE-BNIP3 Normoxia; OE-BNIP3 Hypoxia. A498 and 786-O cells were subjected to an additional experiment involving the introduction of pc-BNIP3, either with or without 3-MA, an autophagy inhibitor, and cultured under hypoxia (OE-BNIP3 + 3-MA Normoxia and OE-BNIP3 + 3-MA Hypoxia groups). Each experiment was performed in triplicate.

### 2.2 Real-time fluorescent quantitative polymerase chain reaction assay

The Trizol Total RNA Extraction Kit (9108, TaKaRa, Japan) was utilized to extract the total cellular RNA, followed by reverse transcription into cDNA using the Reverse Transcription Kit (2690S, TaKaRa, Japan). SYBR Premix Ex TaqTM II kit (RR037Q, TaKaRa, Japan) was used for real-time fluorescence quantification. The real-time qPCR system was performed in a 20 µL system under the conditions of 95°C for 30s pre-denaturation, 95°C for 5s, 55°C for 30s, 45 cycles, and 72°C for 30s extension. The comparative CT method (2^-ddCT^ method) was used to calculate BNIP3 and HIF-1α mRNA expression with β-actin as the internal reference control. Primers were designed using Primer 5.0 Fast software and the sequences are shown in **Table 1** as follows.

**Table 1:** Primers used in this study.

Primer	Forward primer(5’→3’)	Reverse primer(5’→3’)
β-actin	tgacttcaacagcgacaccca	caccctgttgctgtagccaaa
BNIP3	agggctcctgggtagaact	ctccattataaatagaaaccgaggc
HIF-1α	tgctcatcagttgccacttccac	caccagcatccagaagtttcctcac

### 2.3 Cell Counting Kit-8 assay

Cell proliferation was quantified with the Cell CCK-8 kit (BS350B, Biosharp, Shanghai, China), following the manufacturer’s guidelines. The renal carcinoma cells that underwent transfection were plated and left to incubate for a duration of 24 hours. Following this, 10 µL of CCK-8 reagent was added to each well, extending the incubation for three hours. Absorbance at 450 nm was then measured using a microplate reader (ELx800, Buchi Instruments, USA).

### 2.4 Cell cloning assay

Cells were transfected using a lentiviral vector overexpressing BNIP3. The BNIP3 overexpression lentiviral vector was purchased from GeneChem (Shanghai, China). The cell concentration was adjusted to 2 × 10^5^/mL 100 µL of cell suspension was inoculated into each well and the culture medium was replenished. The cells were mixed well and incubated until clones were visible. When clones first became visible, the culture medium was disposed of and the plate was rinsed with PBS. The cells were treated with a 4% volume of polymethanol for a duration of 30 minutes and subjected to staining using a solution containing 0.1% crystal violet for a period of 15 minutes. The plate was dried after removing the staining solution. Photographs were taken, images were recorded, the number of clones visible was counted, and the results were analyzed.

### 2.5 Transwell assay

After trypsin digestion of transfected cells, a 1 × 10^5^ cells/mL concentration was achieved with the medium. Before the experiment, matrix gels were preincubated in Transwell chambers at 37°C for 3 hours. Post-solidification of the Matrigel, serum-free medium was replenished. A cell suspension was then added to Transwell chambers in a 24-well plate, with culture solution outside. The Transwell was washed with PBS after 24 hours of incubation. Fixation was performed using 4% paraformaldehyde, followed by crystal violet staining for 30 minutes. Six random visual fields were selected from each well, and the number of invasive cells was observed with a light microscopic camera system (DMI1, LEICA, Germany).

### 2.6 Apoptosis assay

The A498 and 786-O cells in the logarithmic growth phase were inoculated into 12-well plates at a density of 1×10^5^ cells per well. Once the cells had adhered to the plate, transfection or intervention was carried out. After 48 hours, all cells from the 6-well plates were collected and washed with PBS to eliminate any background interference. The cells were then resuspended in 100 µl of 1×Binding Buffer and incubated with AnnexinV-FITC and PE-PI dual fluorescent probes (KGA1107, KeyGEN, China) for a duration of 15 minutes at room temperature under light protection. Subsequently, the samples were immediately analyzed using flow cytometry (Cytoflex, Beckman Coulter, USA) through the FITC/PI channel to determine the percentage of apoptotic cells.

### 2.7 Western blot analysis

The extraction of total protein from tissues and cells involved the use of lysis buffer, followed by a 30-minute incubation on ice to collect the cells. The supernatant was collected, and the protein concentration was determined by the BCA (P0009, Beyotime, Shanghai, China) method. A 30 μg protein sample was taken and denatured in a boiling water bath and uploaded, proteins were separated by 10% SDS-PAGE and transferred to a PVDF membrane (ISEQ00010, Sigma-Aldrich, USA), and blocked with 5% skimmed milk for 1h at room temperature. The BNIP3 antibody (1:1000, A19593, ABclonal, USA), HIF-1α antibody (1:1000, A7684, ABclonal, USA), caspase3 antibody (1:500, 9662, Cell Signaling Technology, USA), cleaved caspase3 antibody (1:1000, 9661, Cell Signaling Technology, USA), Bax antibody (1:1000, A0207, ABclonal, USA), LC3B antibody (1:1000, A19665, ABclonal, USA), p62 antibody (1:1000, A19700, ABclonal, USA), and β-actin antibody (1:50000, AC026, ABclonal, USA) were incubated at 4°C overnight. The membrane was washed 3 times with TBST, incubated with HRP-labeled secondary antibody (1:7000, S0001, Affbiotech, Jiangsu, China) for 1h at room temperature, washed 3 times with TBST, and developed dropwise with a chemiluminescent solution. Quantitative analysis was performed using Image-ProPlus 6.0 software.

### 2.8 Co-immunoprecipitation

Pretreated cells were lysed in a lysis buffer containing 50 mM Tris-HCl (pH 7.5), 100 mM NaCl, 1% Triton X-100, 0.1 mM EDTA, 0.5 mM MgCl_2_, and supplemented with 10% glycerol, protease inhibitor cocktail (Roche), phosphatase inhibitor cocktail (Roche), and 10 μM pervanadate (NEB). The cell lysates were incubated with Bc1–2 antibody (diluted at a ratio of 1:50; A0208, ABclonal, USA) for one hour at a temperature of 4°C followed by overnight incubation with Protein-A/G Sepharose beads (Abcam, UK) also at a temperature of 4°C. The agarose beads were then collected through centrifugation at a speed of 500 g for five minutes. Subsequently, the beads were thoroughly washed three times in the lysis buffer and heated to a temperature of 95°C for five minutes. Finally, proteins were probed using antibodies against Beclin1(1:30, A7353, ABclonal, USA) and BNIP3 (1:30, A19593, ABclonal, USA).

### 2.9 Immunofluorescence staining

The cell crawl sections were stained. The sections were immersed in 5% film breaker for 10 min. After PBS washing, goat serum sealing solution was added and sealed for 20 minutes at room temperature. Primary antibodies (1:100, LC3B, 14600-1-AP, Proteintech, USA) were added and incubated overnight at 4°C. After washing again with PBS, the second antibody (1:100, FITC-labeled goat anti-rabbit, GB22303, Servicebio, Wuhan, China) was added and incubated for 30 min. After washing with PBS, 4’,6-diamidino-2-phenylindole (DAPI) was added and incubated. After a final wash with PBS, the slices were sealed with an anti-fluorescence attenuating sealer. The images were a3-MAuired using a microscopic camera system (OlyVIA, OLYMPUS, Tokyo, Japan). The fluorescence intensity and area of all images were measured using ImageJ6 (National Institutes of Health, Bethesda, MD, USA), and the mean fluorescence intensity of each image was calculated.

### 2.10 Flow cytometry for ROS

A498 and 786-O cells were cultured in 6-well plates, and a total of 1 × 10^5^ cells from each group underwent two washes with PBS. The cells were then loaded with probes, resulting in a suspension containing 5 mol/L DCFH-DA (MCE). After incubating for 30 minutes at 37°C in the absence of light, the cells were washed twice with PBS. The fluorescence intensity detected using the FITC channel was utilized to quantify the variations in ROS levels among different experimental groups.

### 2.11 Mitochondrial membrane permeability transition pore concentration assay

We used Human MPTP ELISA KIT (ZC-56711, ZCIBIO Technology, Shanghai, China) to detect changes in mitochondrial MPTP concentration. The target antibody is immobilized on a 48-well microplate to form a solid phase carrier. Standard or sample solutions are added to the wells, and the target is connected to the immobilized antibody on the solid phase carrier. Then, horseradish peroxidase-labeled antibody is added, and the unbound antibody is washed away before adding substrate for color development. The absorbance (OD value) at 450 nm is measured using a microcoder (SpectraMAX Plus384, MOLECULAR DEVICES, Shanghai, China), and the sample concentration is calculated.

### 2.12 Tumor xenograft assay

Twelve 6-week-old BALB/c nude mice (18~22 g) were used for subcutaneous tumor xenograft experiments. The animals in this study were purchased from Chengdu Dashuo Experimental Animal Co., Ltd. (Chengdu, China) (SCXK(Chuan)2019-031). BALB/c nude mice were randomly divided into two groups (OE-NC group and OE-BNIP3 group, n =6). In the subcutaneous carcinogenic experiment, the right axillas of nude mice in the OE-NC group and OE-BNIP3 group were subcutaneously injected with stably transfected pcDNA-NC 786-O and pcDNA-BNIP3 786-O cells. The density of 786-O cells was adjusted to 1.0 × 10^6^ cells/mL, and the right axillary side of nude mice was injected subcutaneously to construct tumorigenesis of nude mice for 30 days. Starting on day 20, the tumor volume was measured every 3 days for a total of 10 measurements. After 26 days, the mice were anesthetized (sodium pentobarbital, 100 mg/kg, 200-323-9, Sigma-Aldrich, St. Louis, MO, USA) and sacrificed via cervical dislocation, and all 12 subcutaneous tumors were isolated and weighed. Tumor tissue was then stored in a –80°C freezer for subsequent studies. The Ethics Committee of 363 Hospital (IRB number: 2022035) approved all experimental protocols conducted in this study.

### 2.13 Hematoxylin and eosin staining assay

After completing the experiment, the mice were sacrificed, and part of the tumor tissue was collected, fixed with 4% paraformaldehyde, embedded in paraffin, and sectioned at 4 μm. After dewaxing and rehydration, the histological morphology was observed with hematoxylin-eosin (H&E, C0105M, Beyotime, Shanghai, China) staining.

### 2.14 Transmission electron microscopy analysis

Tumor tissue samples of mice in each group were fixed with 5% glutaraldehyde for up to 5 hours. After fixation, samples were washed three separate times using neutral phosphate buffer, each wash lasting 10 min intervals. Brain tissue samples were fixed with 0.1 mol/L osmic acid for 3 h and washed three times with phosphate buffer, every 10 min. Gradient dehydration was then performed at 50%, 70%, 80%, 90%, 95%, and 100% alcohol concentrations every 15 minutes. The tissue blocks were embedded in resin for ultrathin sectioning (50~70nm), and then stained with uranyl acetate (1261209, SPI), and the ultrastructure of cells and autophagy were observed under a transmission electron microscope.

### 2.15 Terminal deoxynucleotidyl transferase dUTP Nick-End Labeling assay

TUNEL (Terminal deoxynucleotide transferase (TdT) dUTP notch terminal marker) was used to detect apoptosis in tumor tissue cells. Paraffin sections were dewaxed and hydrated with PBS 3 times. The remaining steps of the procedure are conducted by the TUNEL kit (1168479590, Roche Group). Cells with green nuclei under the light microscope were apoptotic positive cells. Three regions were randomly selected from each mouse brain tissue section, and the number of positive cells was used as the apoptotic index.

### 2.16 Immunohistochemical experiment

After the standard preparation of tumor tissue sections, antigen retrieval was performed using citrate buffers followed by treatment with 3% hydrogen peroxide to suppress endogenous peroxidase activity. Afterward, the sections were securely closed using serum derived from goats (AR1009, BOSTER) and incubated overnight with primary antibodies of BNIP3 (1:400, bs-4239R, Bioss, USA) and Ki67 (1:400, HA721115, HUABIO, Shanghai, China) at 4°C, followed by another round of PBS washing. A secondary antibody (1:100, GB22303, Servicebio, Shanghai, China) was added and incubated at 37°C for 30 min. Tissues were visualized using a DAB (36201ES03, YEASEN) staining solution, and hematoxylin restaining was performed before dehydration and sealing. Image analysis was performed using a digital trimoscope (BA210Digital, Meiji Technology Co., LTD., China) and a data image analysis system (Halo 101-WL-HALO-1, Indica Labs, USA).

### 2.17 Statistical analysis

The statistical analysis was conducted using GraphPad Prism 9.0 software. The mean () ± standard mean (SD) were used to represent the experimental statistics. To compare multiple groups, a one-way analysis of variance (ANOVA) was employed, and statistical analysis was performed *post hoc* by Tukey. The T-test was used for comparison between the two groups. *P*<0.05 was statistically significant.

## 3 Results

### 3.1 Expression of BNIP3 and HIF-1α in renal cell carcinoma cell lines

**Figure 1:**
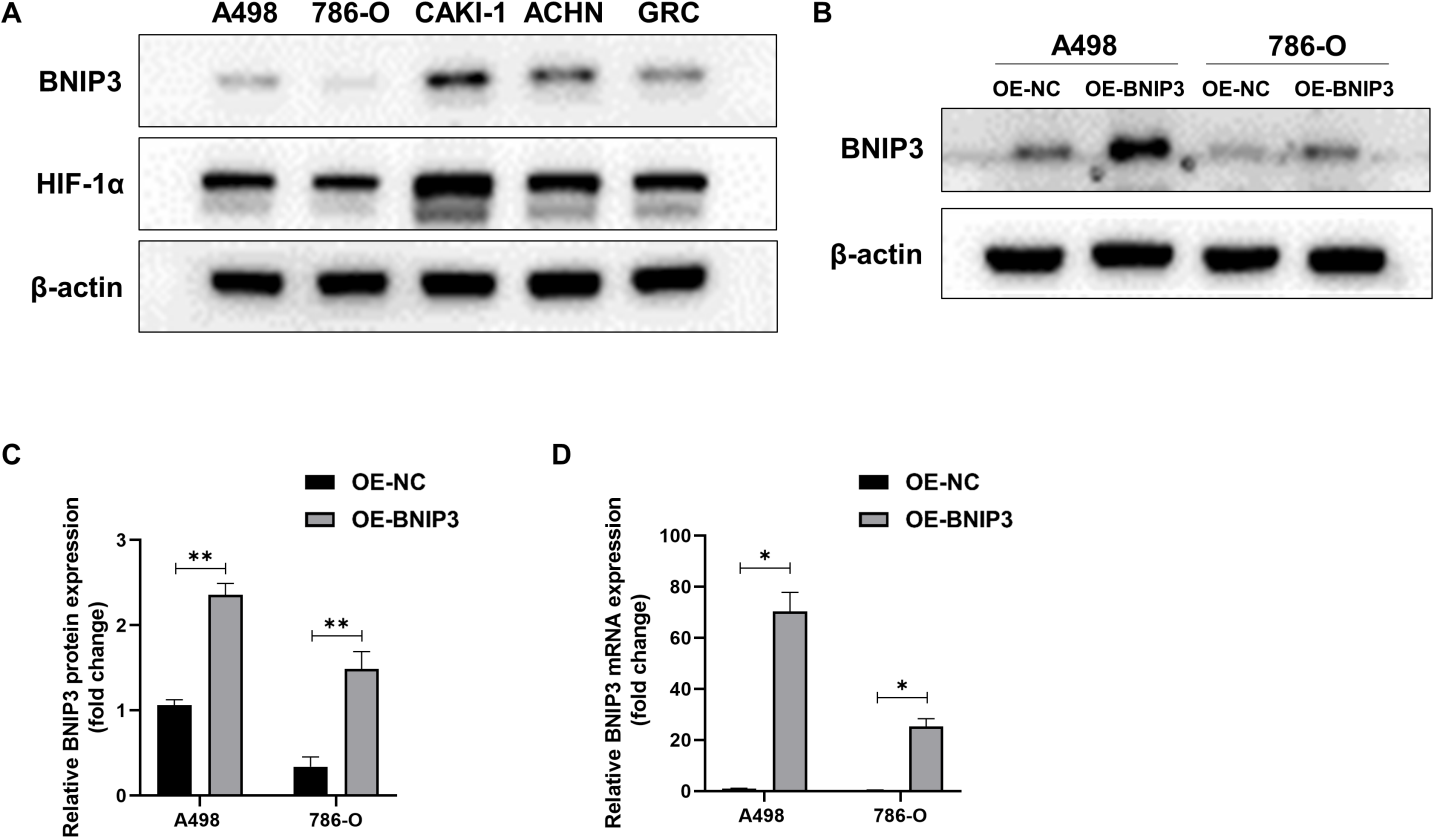
Expression levels of BNIP3 and HIF-1α in renal cell carcinoma (RCC) cell lines. **(A)** Western blot analysis of BNIP3 and HIF-1α expression levels in A498,786-O, CAKI-1, ACHN, and GRC cell lines. **(B, C)** Western blot analysis of BNIP3 expression levels in A498 and 786-O cell lines. **(D)** mRNA expression levels of BNIP3 in A498 and 786-O cell lines were examined using reverse transcription-quantitative PCR. Data are mean ± SD, *P < 0.05, **P < 0.01.

Initially, we investigated the protein expression of BNIP3 and HIF-1α in renal cell carcinoma cell lines A498, 786-O, CAKI-1, ACHN, and GRC. As depicted in **Figure 1A**, elevated levels of BNIP3 and HIF-1α were observed in CAKI-1, ACHN, and GRC, whereas these proteins exhibited minimal expression in A498 and 786-O. Consequently, A498 and 786-O were chosen as the subjects of investigation. WB and RT PCR were used to detect the expression of BNIP3 protein and mRNA in A498 and 786-O cells with overexpression of BNIP3. As shown in **Figures 1B, C**, the level of BNIP3 protein expression was notably elevated in the OE BNIP3 group (BNIP3 expression group) compared to the OE-NC group (negative control group). Furthermore, BNIP3 mRNA was significantly increased in the OE BNIP3 group compared to the OE-NC group (*P*<0.01) (**Figure 1D**).

### 3.2 Effect of overexpression of BNIP3 on the proliferation, invasion, and apoptosis of renal cell carcinoma cells

**Figure 2:**
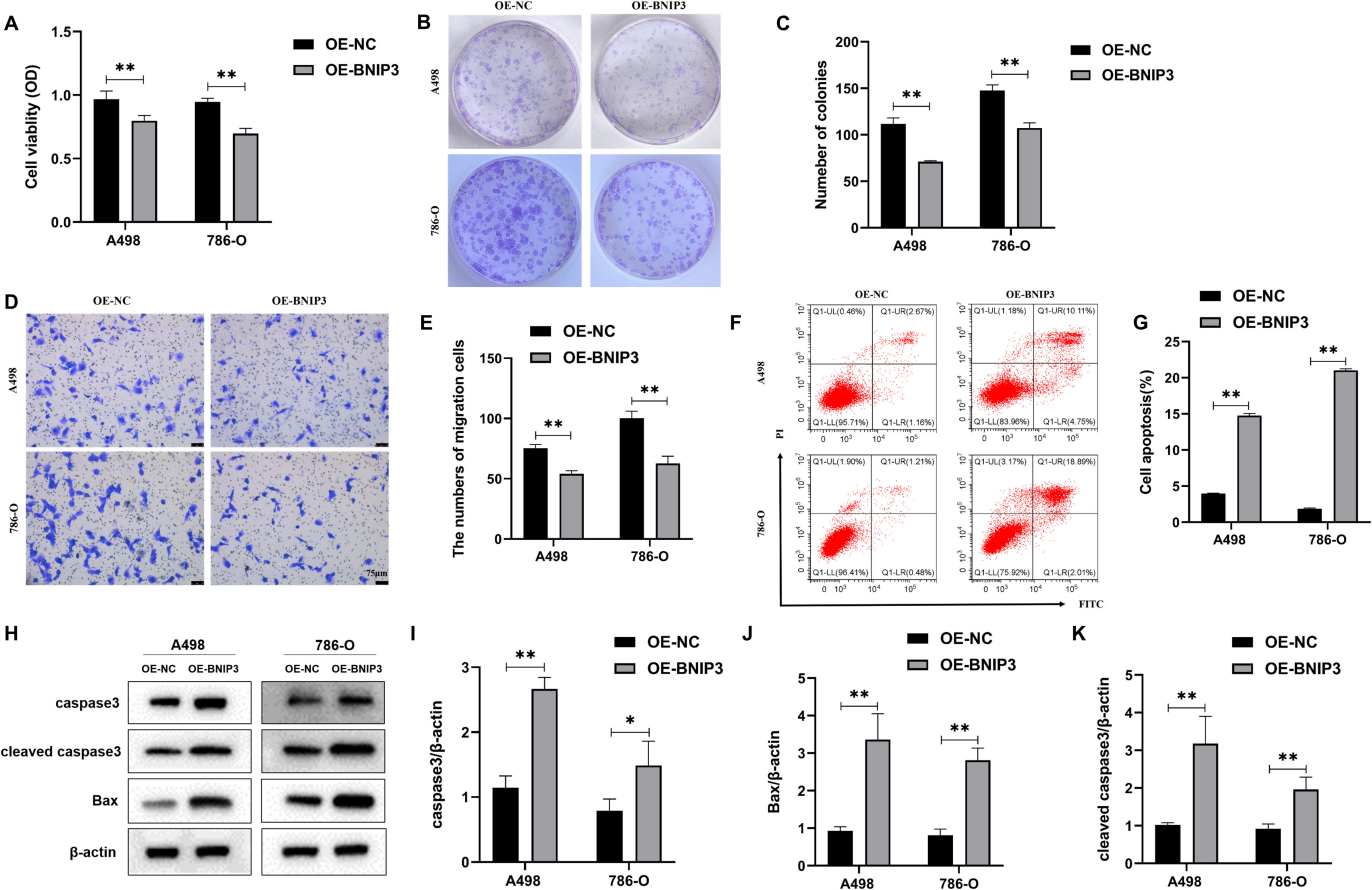
The impact of overexpression of BNIP3 on renal cell carcinoma (RCC) cells’ proliferative activity, invasion, and apoptosis ability. **(A)** Results of cell counting kit-8 (CCK-8) assay to detect proliferation activity. **(B, C)** Plate clone formation assay to detect the ability of RCC cells to form clones. **(D, E)** Transwell assay for assessing the invasiveness of RCC cells (×40). **(F, G)** Revealing cell apoptosis in different cell groups using flow cytometry. **(H-K)** Western blot detection of caspase3, cleaved caspase3, and Bax. Data are mean ± SD, *P < 0.05, **P < 0.01.

A498 and 786-O cells were transfected and divided into the OE-NC and OE-BNIP3 groups. According to the data presented in **Figure 2A**, there was a significant decrease in the proliferative activity of the OE-BNIP3 cells compared to that of the OE-NC group. We obtained comparable findings from the cell cloning assay, with the number of clones formed by A498 and 786-O cells in the OE-BNIP3 group being significantly less than that in the OE-NC group (*P*<0.01) (**Figures 2B, C**). Furthermore, the Transwell invasion assay indicated that the OE-BNIP3 group had a significantly lower number of A498 and 786-O cells crossing the Transwell compartment than the OE-NC group (*P*<0.01) (**Figures 2D, E**). Flow cytometry showed that OE-BNIP3 had increased A498 and 786-O cell death compared to OE-NC (*P*<0.01) (**Figures 2F, G**). In addition, OE-BNIP3 increased the expression of caspase3, cleaved caspase3, and Bax compared with OE-NC (**Figures 2H–K**). Based on the above results, overexpression of BNIP3 suppressed the proliferation and invasion and promoted apoptosis in renal cell carcinoma cells.

### 3.3 Overexpression of BNIP3 promotes the dissociation of the Bcl-2/Beclin1 complex through competitive binding of Bcl-2 under hypoxia conditions

**Figure 3:**
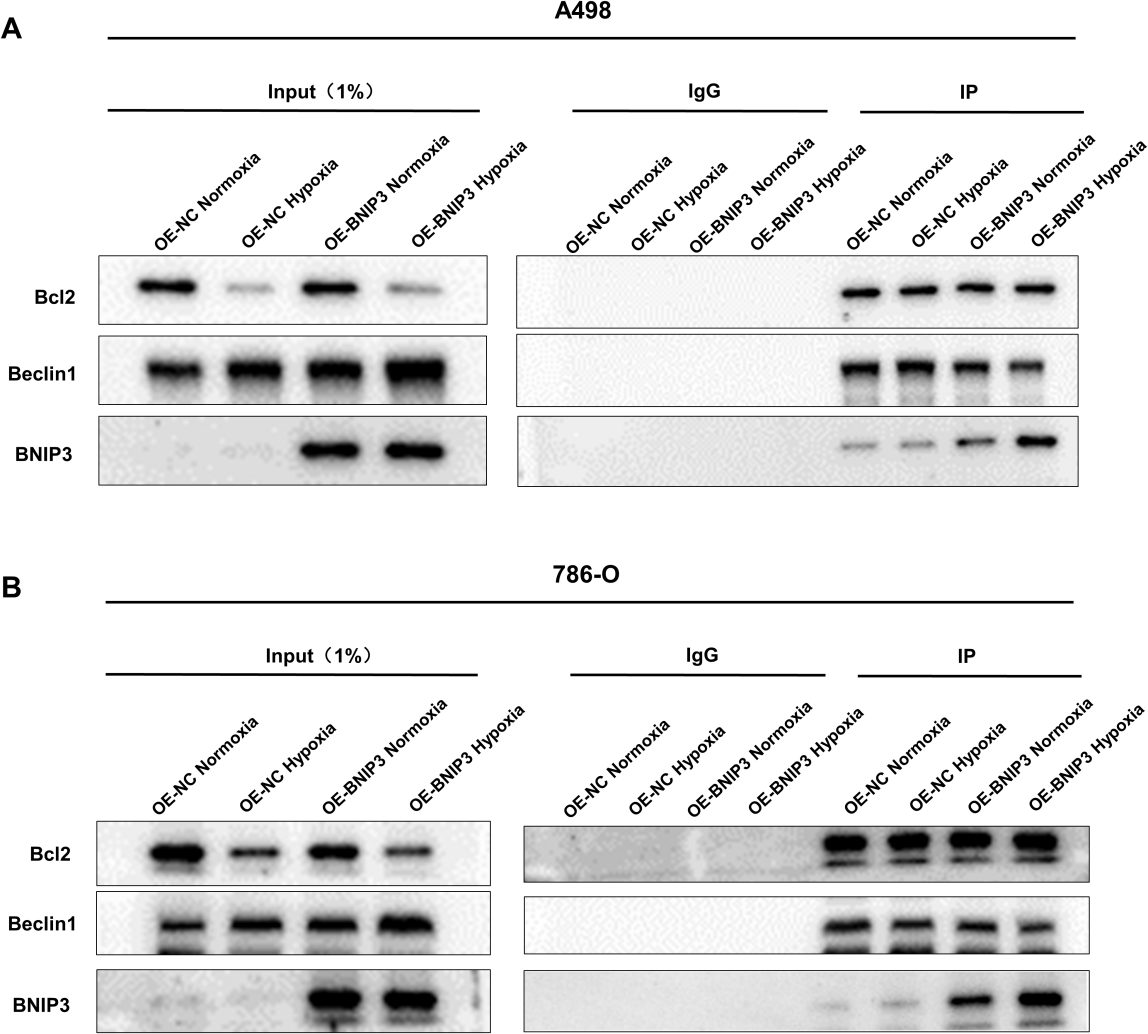
Overexpression of BNIP3 promotes the dissociation of the Bcl-2/Beclin1 complex through competitive binding of Bcl-2 under hypoxia conditions. **(A, B)** The dissociation of Bcl-2/Beclin1 complex in BNIP3-overexpressing A498 and 786-O cells was indicated by the Co-IP experiment with Beclin1 antibody and Bcl-2 antibody.

In the following step, our objective is to determine whether the overexpression of BNIP3 can definitively bind to BCL-2 and induce the dissociation of Beclin-1 from Bcl-2. By performing co-immunoprecipitation experiments, we successfully co-precipitated Bcl-2. Analysis using the Western Blot technique demonstrated that under hypoxic conditions, BNIP3 overexpression significantly increased the co-precipitation of Bcl-2 with BNIP3, while simultaneously reducing the co-precipitation of Bcl-2 with Beclin1 (**Figures 3A, B**). This indicates an enhanced binding capacity between BNIP3 and Bcl-2, and a weakened binding capacity between Bcl-2 and Beclin1. This change led to the dissociation of the Bcl-2/Beclin1 complex, thereby promoting the process of protective autophagy in RCC cells under hypoxic conditions.

### 3.4 Overexpression of BNIP3 promotes autophagy and mitochondrial damage in renal carcinoma cells under hypoxia

**Figure 4:**
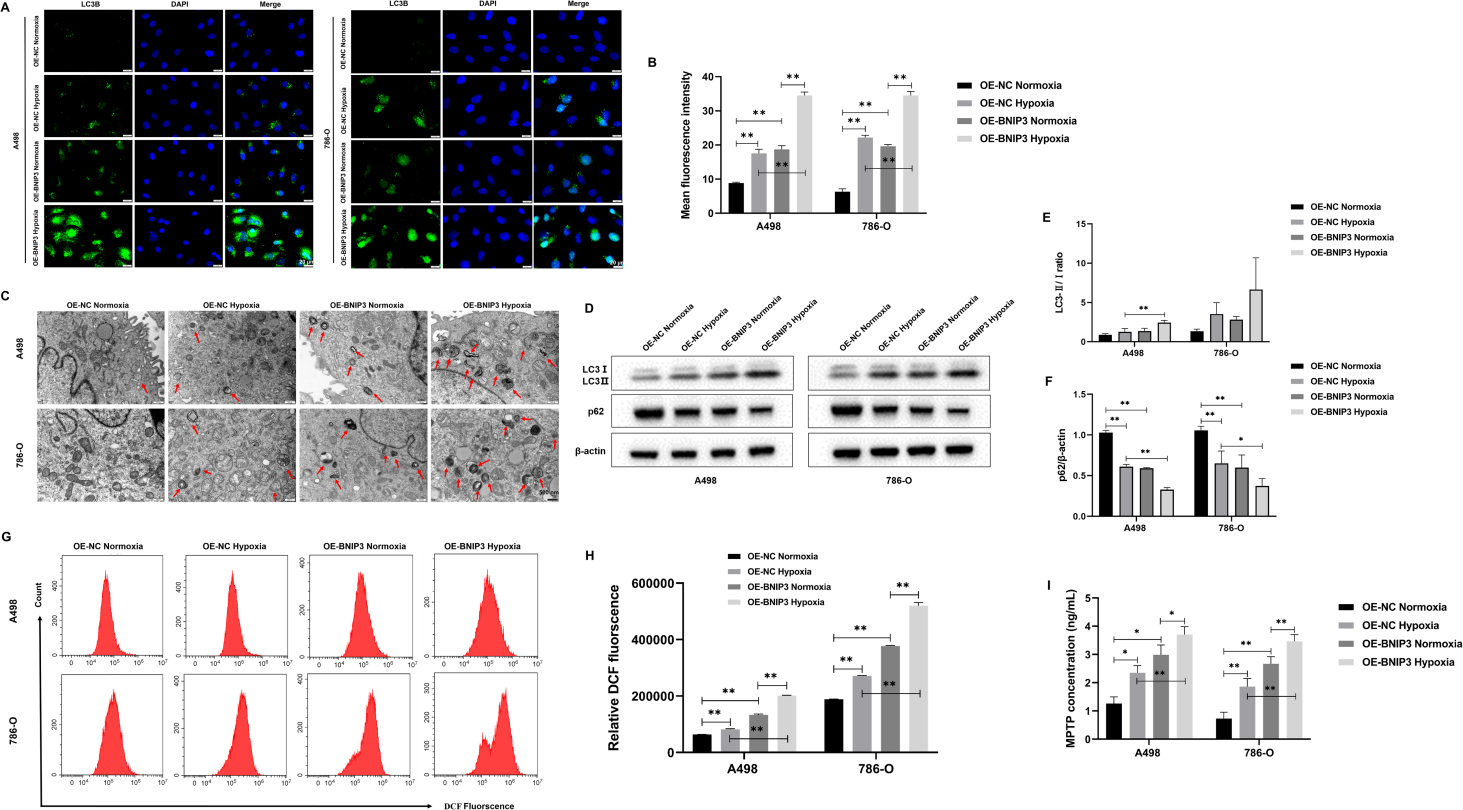
Overexpression of BNIP3 promotes autophagy in renal cancer cells under hypoxia. **(A)** Immunofluorescence staining was used to detect the expression levels of LC3B (×40). **(B)** Mean fluorescence intensity of LC3B. **(C)** The autophagosomes in A498 and 786-O cells were visualized using transmission electron microscopy (TEM) at a magnification of ×20000 after exposure to hypoxia. **(D-F)** Western blot detection of LC3-I, LC3-II, and p62. **(G, H)** Overexpression of BNIP3 affecting A498 and 786-O cells ROS. **(I)** Concentration of mitochondrial membrane permeability transition pore (mPTP). Data are mean ± SD, *P < 0.05, **P < 0.01.

To explore the effect of overexpression of BNIP3 on autophagy under hypoxia. LC3B is a key protein involved in autophagy and is crucial in regulating cellular processes and responses to stress. The results of immunofluorescence staining confirmed that A498 and 786-O cells exposed to hypoxia exhibited elevated levels of LC3B compared to those cultured under normoxic conditions. Moreover, additional treatment with OE-BNIP3 further enhanced the effects induced by hypoxia. (**Figures 4A, B**). In the context of TEM analysis, it was observed that the number of autophagosomes in A498 and 786-O cells exhibited an elevation upon undergoing hypoxia treatment. Notably, the augmentation in the count of autophagosomes was more pronounced in cells that had undergone BNIP3 overexpression (**Figure 4C**). Western blot analysis revealed that when compared to A498 and 786-O cells under normoxic conditions, the expression of p62 in 786-O cells under hypoxic conditions experienced a decrease, and the ratio of LC3-II to LC3-I exhibited an elevation in A498 and 786-O cells. However, the presence of BNIP3 overexpression contributed to the facilitation of these hypoxia-induced effects (**Figures 4D-F**). Under hypoxia conditions, ROS levels increased in A498 and 786-O cells, and overexpression of BNIP3 increased ROS levels compared to the OE-NC hypoxia and the OE-BNIP3 normoxia groups (*P*<0.01) (**Figures 4G, H**). Additionally, under hypoxic conditions, the extent of mitochondrial damage was exacerbated in A498 and 786-O cells, and this damage was further exacerbated upon transfection with BNIP3 overexpression (**Figure 4I**).

### 3.5 Overexpression of BNIP3 promotes apoptosis of renal cancer cells by mediating autophagy under hypoxia

**Figure 5:**
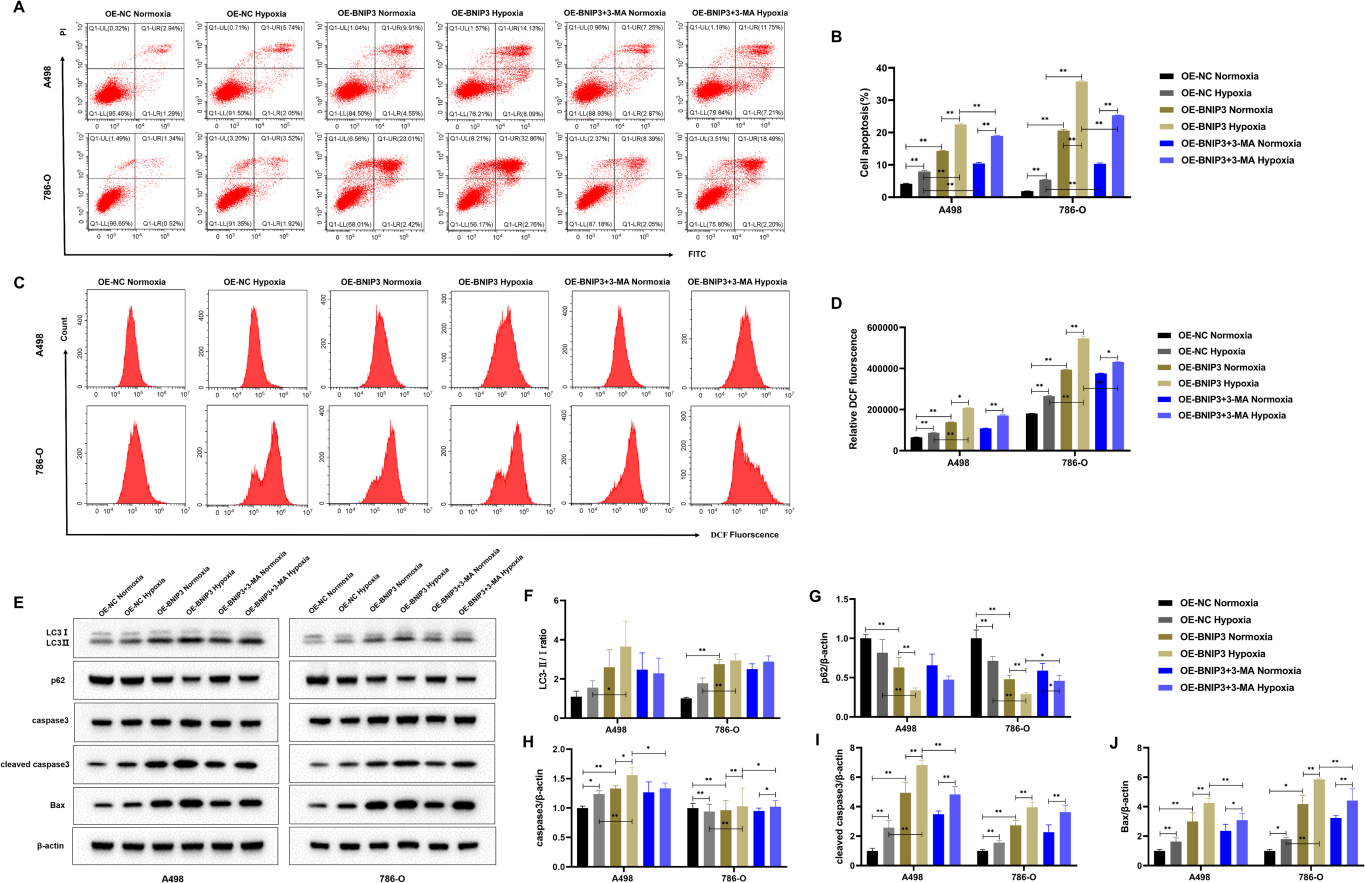
Overexpression of BNIP3 promotes apoptosis of cancer cells by mediating autophagy under hypoxia. **(A, B)** Apoptosis was detected by flow cytometry. **(C, D)** Overexpression of BNIP3 affecting A498 and 786-O cells ROS. **(E-J)** Western blot detection of LC3-I, LC3-II, p62, caspase3, cleaved caspase3 and Bax. Data are mean ± SD, *P < 0.05, **P < 0.01, ^#^ *P* < 0.05, ^##^ *P* < 0.01.

To explore the effect of autophagy inhibitor 3-MA combined with OE-BNIP3 on apoptosis of renal cell carcinoma cells by mediating autophagy under hypoxia conditions. Flow cytometry results showed that BNIP3 overexpression promoted apoptosis in A498 and 786-O cells under hypoxic conditions, and the addition of autophagy inhibitor 3-MA could inhibit this promotion and reduce apoptosis (**Figures 5A, B**). As shown in **Figures 5C, D**, ROS production is more likely to be promoted by BNIP3 overexpression in a hypoxic environment, and the autophagy inhibitor 3-MA can effectively inhibit this promoting effect. The findings from WB analysis indicated that the level of expression for autophagy-related regulator LC3-II/LC3-I increased and p62 protein decreased in the OE-BNIP3 hypoxic group. In contrast, the addition of autophagy regulator 3-MA reversed the expression of LC3-II/LC3-I and p62 proteins (**Figures 5E–G**). For the increase of the protein expression associated with programmed cell death cleaved caspase3 and Bax in the OE-BNIP3 hypoxic group, the addition of autophagy regulator 3-MA had a certain inhibitory effect, and the protein expression of cleaved caspase3 and Bax in the OE-BNIP3 + 3-MA hypoxic group decreased (**Figures 5E, H-J**). Based on the above results, it was found that BNIP3 overexpression promoted the apoptosis of renal cancer cells by mediating autophagy under hypoxic conditions.

### 3.6 Overexpression of BNIP3 inhibits tumor growth in tumor-bearing mice

**Figure 6:**
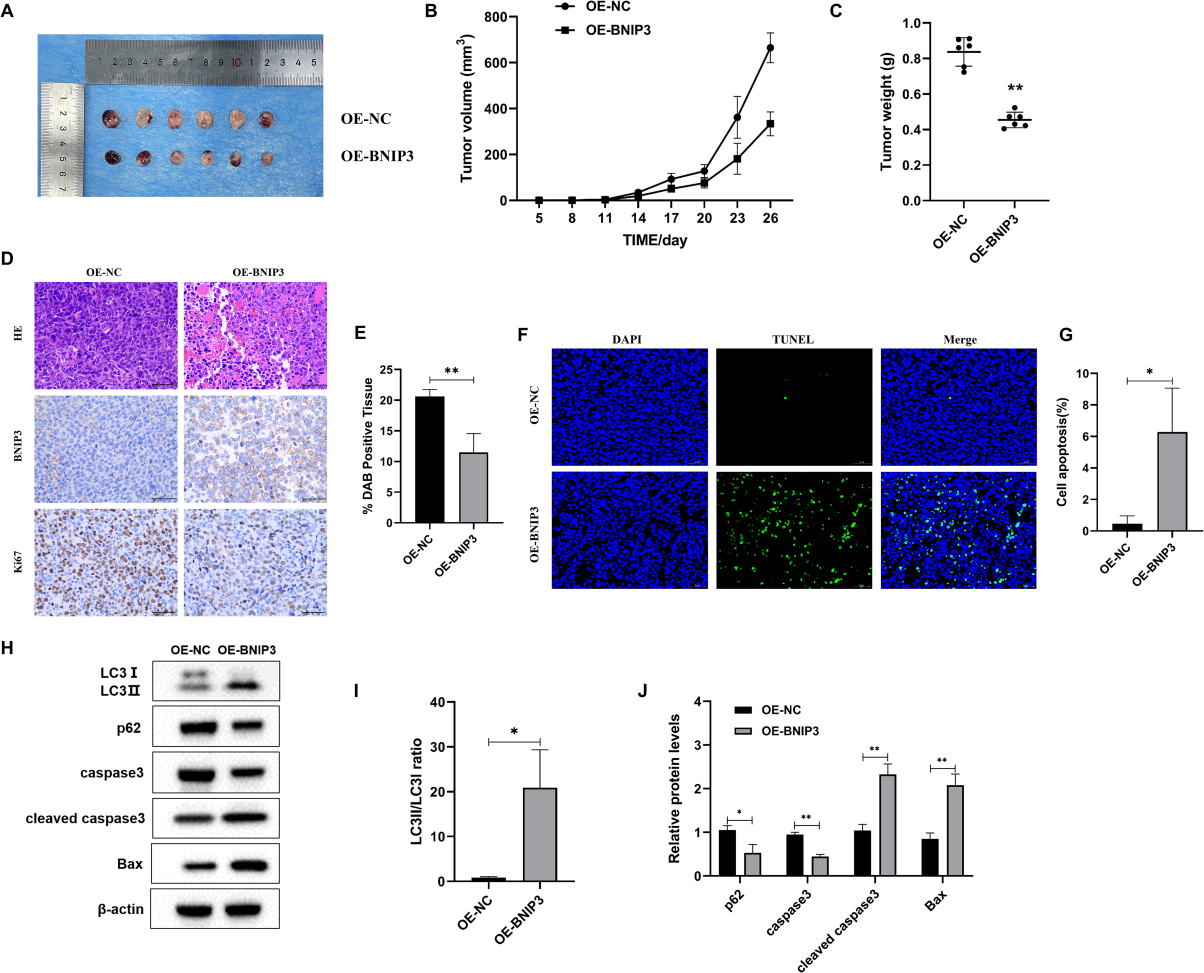
Overexpression of BNIP3 suppresses tumor growth in mice with established tumors. **(A)** Tumor plot of dissected xenograft tumors. **(B)** Tumor volume growth curve. **(C)** Tumor weight. **(D, E)** Microscopic observation of HE and immunohistochemical detection of ki-67 expression level of tumors (×40). **(F, G)** Apoptosis of tumor tissue was detected by TUNEL staining (×40). **(H-J)** Western blot detection of LC3-I, LC3-II, p62, caspase3, cleaved caspase3, and Bax proteins. Data are mean ± SD, *P < 0.05, **P < 0.01.

After 26 days, the mice were euthanized under anesthesia via cervical dislocation, and all 12 subcutaneous tumors were removed and their weights were measured (**Figure 6A**). The tumor sizes in each group of mice were measured. The OE-NC group experienced a significantly faster increase in tumor volume (**Figure 6B**). Upon completion of the experiments, the dissected and isolated tumors were weighed. In comparison to the OE-NC group (**Figure 6C**), the OE-BNIP3 group showed notably reduced tumor volume. Meanwhile, the OE-BNIP3 group exhibited significantly reduced levels of Ki67 expression in tumor tissues compared to the OE-NC group (**Figures 6D, E**). The Tunel staining technique was employed to assess tumor cell apoptosis, revealing a significant increase in apoptosis within the OE-BNIP3 group (**Figures 6F, G**). In addition, the protein expression levels of LC3-II/LC3-I, caspase 3, cleaved caspase 3, and Bax were found to be significantly elevated in the OE-BNIP3 group compared to the OE-NC group. Conversely, the protein expression levels of p62 were observed to be lower in the OE-BNIP3 group as compared to the OE-NC group (**Figures 6H-J**). This part of the experiment showed that overexpression of BNIP3 can inhibit tumor growth *in vivo* and promote the occurrence of autophagy and apoptosis of tumor cells.

## 4 Discussion

RCC arises from the epithelial cells of the renal tubules and is among the frequently occurring malignant tumors within the urinary system, with an incidence of about 3% of all malignant tumors and a mortality rate of 30~40% (16, 17). Although targeted therapies (e.g., tyrosine kinase inhibitors, vascular endothelial growth factor inhibitors, and mTOR inhibitors) and new immunotherapy strategies have been applied to some clinical treatments, the heterogeneity of RCC, the relative resistance to radiotherapy and chemotherapy, and other side effects make it difficult to achieve the expected efficacy, so it has become a top priority to explore new treatment strategies and intervention options (18).

The majority of clear cell renal cell carcinomas (ccRCCs) are triggered by the accumulation of HIF and the overexpression of downstream genes as a response to the inactivation of the von Hippel-Lindau (VHL) gene. BNIP3, a pro-apoptotic member of the Bcl-2 family proteins, contains a hypoxia response element (HRE) in its gene promoter directly regulated by the HIF (19, 20). The expression of BNIP3 in different tumors is inconsistent, and its high expression in tumors such as lung cancer, prostate cancer, breast cancer, and endometrial cancer is associated with a bleak prognosis for patients diagnosed with tumors. Still, its level of expression is minimal in cases of pancreatic cancer, colorectal cancer, liver cancer, and other tumors, and is associated with drug resistance in tumor patients (21). At present, a comprehensive analysis of the expression and function of BNIP3 in ccRCC is unavailable. On this basis, the expression of BNIP3 protein and mRNA in renal cell carcinoma cell lines A498, 786-O, CAKI-1, ACHN, and GRC was analyzed, and A498 and 786-O cells with lower expression were selected to be transfected with BNIP3 overexpression plasmid to construct overexpressing BNIP3. Additionally, the spread and infiltration of cancer cells are defining traits of tumor cells, and the metastasis of tumors is a multifaceted procedure that encompasses cell movement, infiltration, and attachment (22). The previous studies have demonstrated that treatment with TSA can restore the acetylated state of the BNIP3 gene, enhance the expression levels of both BNIP3 mRNA and protein, suppress cell proliferation, and induce cell death in RCC (8). The outcomes of this experimental investigation revealed that the overexpression of BNIP3 led to a notable reduction in cell survival and a diminished capacity for both colony formation and cell migration across RCC cell lines, thereby suggesting that BNIP3 overexpression has the potential to suppress the proliferative and metastatic capabilities of renal cancer cells. Moreover, the apoptosis assays demonstrated that the upregulation of BNIP3 significantly accelerated the process of apoptosis in A498 and 786-O cell lines, and concurrently elevated the expression levels of key apoptosis marker proteins, including caspase3, cleaved caspase3, and Bax.

Autophagy constitutes a cellular mechanism of self-degradation, wherein lysosomes are employed to break down impaired or unfolded macromolecular components or organelles in response to extrinsic environmental stimuli (23). The Bcl-2/Beclin1 complex is a major autophagy modulator (24). The ULK1 complex is required for autophagy initiation, and the assembly of the Bcl-2/Beclin1 complex prevents Beclin-1 from activating ULK1, thereby initiating the autophagy process (25). Studies have shown that BNIP3 can promote the occurrence of autophagy by releasing Beclin1, which may be related to preventing Bcl-2 from binding to Beclin1 to activate autophagy (26). BNIP3 not only activates cell apoptosis but also induces autophagy that promotes survival under certain conditions, and the choice of this function depends on the specific environment of the cell (27). Under hypoxic conditions, the upregulation of BNIP3 expression promotes autophagy through competitive binding with Bcl-2 for Beclin1, thereby facilitating the release of Beclin1 and subsequent initiation of autophagic processes (28). The functional mechanism of BNIP3 involves its competitive interaction with Bcl-2, specifically engaging Bcl-2 via its BH3 domain, which inhibits the formation of the Bcl–2–Beclin1 complex and subsequently facilitates the induction of autophagy (29). Studies have shown that the expression level of BNIP3 in RCC tumor tissues is low, and overexpression of BNIP3 will promote autophagy in RCC cells (30). Current evidence also indicates that overexpression of BNIP3 can affect the autophagic process through multiple pathways (31, 32). In this study, CO-IP experiments showed that the overexpressed BNIP3 competitively binds to Bcl-2 and dissociates Beclin1, triggering autophagy. Under hypoxic conditions, the oxygen content in the cells decreases and energy metabolism is affected, at which point autophagy is activated to help the cells cope with energy crises and metabolic stress (33). Hypoxia has been reported to activate autophagy, accompanied by increased BNIP3 expression (10). This experiment found that the dissociation ability of the Bcl-2/Beclin1 complex was stronger under the promotion of overexpression of BNIP3 under hypoxic conditions, which was consistent with the above conclusions. Notably, a comparable trend was observed under normoxic conditions, which may be attributed to the presence of basal cellular stress states or the constitutive activation of endogenous signaling pathways. Despite being maintained in a normoxic environment, cells exhibit intrinsic metabolic activities and dynamic redox regulation that can influence the interactions among autophagy-related proteins. For example, low levels of endogenous oxidative stress may persist, which could partially activate autophagy-associated signaling cascades, thereby promoting the association between Bcl-2 and BNIP3 while attenuating the binding of Beclin1 to Bcl-2.

BNIP3 is a protein localized to the outer membrane of mitochondria, acting as an autophagy receptor, and is thought to be an important signaling molecule for hypoxia-induced autophagy (34). The HIF-1α/BNIP3 pathway is a key mechanism regulating autophagy, in which HIF-1α plays a key role in hypoxia-induced autophagy, and BNIP3 acts as a direct downstream regulator of HIF-1α under hypoxic conditions, promoting the expression of BNIP3 and thereby inducing the occurrence of autophagy (35). LC3, a protein associated with microtubules and known as light chain 1 of microtubule-associated protein 1, acts as a marker for autophagy and is present on lysosomal and mitochondrial autophagosome membranes (36). LC3B, a well-established marker of autophagy, has been extensively utilized in numerous studies to evaluate the activation status of autophagy through its expression levels (37). Upon autophagy induction, LC3B undergoes a critical transformation from its cytosolic form (LC3B-I) to the membrane-bound form (LC3B-II) (38). This conversion represents an essential step in the expansion and maturation of autophagosome membranes, thereby playing a pivotal role in the progression of autophagy. During autophagy, LC3 protein is cleaved by autophagic protein 4 (Atg4) to produce LC3-I, which is subsequently processed and modified by a ubiquitin-like system to form LC3-II, and the content of LC3-II is directly proportional to the degree of autophagy (39). BNIP3 can bind directly to LC3 to induce the occurrence of autophagy (40). The research findings suggest that the knockdown of LBX2-AS1 can enhance autophagy by upregulating BNIP3L levels and curbing the proliferation of ccRCC cells (41). Our results suggest that overexpression of BNIP3 under hypoxic conditions induces an increase in LC3B levels, an increase in autophagosomes, an increase in LC3-II protein expression, a decrease in p62 protein expression, and an enhancement of autophagy. In addition, the potential of overexpression of BNIP3 to suppress cancer and promote autophagy has also been demonstrated in nude mice.

Apoptosis is the mode of programmed cell death, in which the mitochondrial permeability transition pore (mPTP) opens up and the mitochondrial membrane potential (MMP) decreases when stimulated by intracellular signaling, and mitochondrial damage leads to the activation of autophagy (42). At the same time, the reduction of MMP can swell the mitochondrial matrix, release cytochrome C and apoptosis-inducing factors into the cytosol, and initiate cysteine aspartate-specific protease (caspase)-dependent or independent apoptosis (43). The depolarization of MMP is a common process between autophagy and apoptosis initiation, suggesting that there is an internal correlation between the two. Studies have shown that key proteins involved in autophagy, such as PINK1, Parkin, BNIP3, and NIX, are important intermediates of various physiological processes in cells, and the genes encoding these proteins are tumor suppressor genes that promote apoptosis (44, 45). In addition, aberrant autophagy can disrupt mitochondrial homeostasis, thereby inducing apoptosis (46, 47). Studies have demonstrated that silencing the expression of BNIP3 and BNIP3L/NIX completely inhibits autophagy induction by hypoxia in CCL39 cells and renders them pro-apoptotic proteins (10). Similarly, BNIP3 induces mitochondrial dysfunction and promotes autophagy and apoptosis in neonatal cardiac myocytes under hypoxia (48). In this study, overexpression of BNIP3 promoted autophagy to disrupt mitochondrial homeostasis and induced cell apoptosis under hypoxic conditions, while adding an autophagy inhibitor 3-MA reduces apoptosis, indicating that BNIP3 overexpression-induced cell apoptosis is autophagy-dependent.

## 5 Conclusion

In conclusion, our study has demonstrated that the overexpression of BNIP3 effectively suppresses the proliferation, cloning, and migratory capacity of RCC cells while inducing apoptosis. Moreover, overexpression of BNIP3 in hypoxic conditions induces autophagy by disrupting the interaction between BCL-2 and Beclin1, thereby facilitating apoptosis in RCC cells through the regulation of autophagy. Therefore, this study contributes to identifying the impact of BNIP3 overexpression on cell proliferation and apoptosis in hypoxia-induced autophagy in RCC cells, offering a novel therapeutic target for impeding RCC progression.

## Data Availability

The original contributions presented in the study are included in the article/supplementary material. Further inquiries can be directed to the corresponding author.
